# Conditions to eliminate cholera in Mozambique - the pathway for the development of the national cholera plan

**DOI:** 10.11604/pamj.2022.42.279.36368

**Published:** 2022-08-15

**Authors:** Cynthia Semá Baltazar, Lorenzo Pezzoli, Liliana Dengo Baloi, Naira Luiz, Jucunu Elias Chitio, Igor Capitine, Major Sitoe, Simões Mala, José Paulo Langa

**Affiliations:** 1 Instituto Nacional de Saúde, Maputo, Mozambique,; 2 International Federation of Red Cross and Red Crescent Societies, Country Support Platform, Geneva, Switzerland

**Keywords:** Cholera, Mozambique, national cholera plan (NCP), elimination, challenges

## Abstract

Cholera disproportionately affects the most vulnerable segments of the population, particularly those who have low or no access to basic water, sanitation, and hygiene (WASH). Despite some improvements in WASH conditions, cholera still represents a persistent challenge in Mozambique, where outbreaks occur almost every year, with high case fatality rates, posing a threat to the country's economic development. The Government of Mozambique has started developing a revised National Cholera Plan (NCP), which aligns with “ending cholera-a global roadmap to 2030” launched by the Global Task Force on Cholera Control (GTFCC) in 2017. Ending cholera represents a critical step towards achieving the sustainable development goals and requires effective prevention and control interventions, ensuring that no one is left behind. The NCP must use a multi-sector approach and broad stakeholder collaboration with well-coordinated roles and functions of different partners to address major areas for cholera elimination - water and sanitation, health care services and management, epidemiology and surveillance, and health and hygiene promotion. Every cholera death is preventable. In this review, we reiterate the need for effective coordinated actions to control and eliminate cholera in Mozambique and decrease the cholera burden, enabling a healthy population over the generations.

## Commentary

Known as the “disease of poverty”, or the “disease of inequality”, cholera is an acute waterborne diarrheal disease caused by the bacterium *Vibrio cholera*, mainly transmitted by the ingestion of contaminated food or water. When untreated, cholera can lead to death due to severe dehydration. Although it is a preventable and treatable disease that can be realistically eliminated as a public health threat, cholera remains a significant problem in many low-and middle-income countries, because of inadequate access to clean water, sanitation, and hygiene (WASH), poor infrastructure, and fragile health systems that lead to recurrent outbreaks [1-3]. Annually around 1.4 to 4.0 million cholera cases with nearly 143.000 deaths occur worldwide, and most sub-Saharan countries affected by cholera are classified as endemic, with most cases occurring during rainy seasons [4]. Large cholera outbreaks are usually preceded by complex emergency contexts, including armed conflicts, natural disasters, migration crises, and others. Those crises often result in population displacement, with increased risk of overcrowding, inadequate water supplies, poor sanitation, and hygiene conditions, scarce resources, and usually a worsening of the health system as a whole. All these factors increase the risk and severity of cholera outbreaks.

Despite experiencing recurrent outbreaks over the years, the sub-Saharan African region has achieved limited success in preventing and controlling cholera. Data suggests that the sub-Saharan African region has accounted for the majority of the cholera burden from 2000-2015, with 83% of cholera deaths recorded. It should be noted, however, that the real situation may be worse since data is often underreported due to lack of diagnostic facilities, weak or absent surveillance systems, and fear of the negative economic impact on the tourism industry and international embargo on commodities exportation [5]. On a global level, attention has been focused in recent decades on developing a multi-sectorial approach to end cholera. In 1992 the Global Task Force on Cholera Control (GTFCC) was established. This is a multi-sectoral network of international agencies, academic institutions, and non-governmental organizations (NGOs) from around the world that supported countries in their fight against cholera, offering an effective and well-coordinated platform whose secretariat is hosted by the WHO. In 2017 the GTFCC launched the strategy “Ending cholera: a global roadmap to 2030”. The global roadmap aims to reduce global cholera deaths by 90% and eliminate the disease in at least 20 countries by 2030 with the commitment of cholera-affected countries, technical partners, and donors [1].

The roadmap is based on three key axes: 1) rapid response to cholera outbreaks; controlling epidemics through community engagement, improved early warning surveillance, and the rapid delivery of cholera control kits, reactive use of Oral Cholera Vaccine (OCV), and ensuring emergency WASH supplies; 2) implementation of a multisectoral approach in hotspots of endemic cholera, implementation of mass preventive campaigns, creation of sustainable WASH infrastructure, strengthening of health systems to anticipate cholera outbreaks, development of strong community engagement through social mobilization strategies for hygiene promotion and risk communication and establishment of cross-border collaboration; 3) coordination of technical support and expertise by GTFCC partners, both locally and globally, supporting countries to develop comprehensive and multisectoral control plans. For effective implementation and scale-up of those interventions to reach this ambitious 2030 target, countries must define strategies to identify and adequately address high-risk populations [1].

Mozambique is one of the sub-Saharan African countries that has been facing recurrent cholera outbreaks [3]. Given the high disease burden in the country and the recent global developments following the launch of the roadmap, the Mozambique government is revising the (NCP), so that it is aligned with the roadmap. The plan's design requires a comprehensive, integrated, and multi-sectorial approach, long-term prevention strategies and ultimately depends on political commitment.

**Cholera epidemiology in Mozambique:** in the current days, cholera in Mozambique is an endemic disease with epidemic peaks. The annual incidences that vary from 0 to 211 per 100,000 population, and case-fatality that varies from 0.2% to 4.3%. The burden of cholera has remained high, and hotspots occur mainly in the central and northern regions; especially in the north provinces [3]. The country is one of the regional countries most vulnerable to climate change. In 2015, a massive cholera outbreak was officially declared, and the situation worsened with extensive flooding, which led to over 8,835 cholera cases reported with a death toll of 65%. In March 2019, Mozambique was severely affected by two tropical cyclones. Categorized as level four, the first - cyclone Idai hit the central part of Mozambique, mainly Sofala Province, which led to more than 80% of facilities damaged including houses and all water distribution infrastructures, with more than 400,000 people displaced and more than 200 deaths. Six weeks after Idai, the country was hit by the second cyclone - Kenneth (level three) in the extreme north of the country, impacting Cabo Delgado Province, affecting nearly 45,000 people and leading to 45 deaths. The Mozambique president declared a state of emergency, and the World Health Organization (WHO) characterized the country's humanitarian situation as grade 3 emergencies. The situation leads to a cholera outbreak in the two provinces. Sofala registered 6768 suspected cholera cases, an attack rate of 571 per 100 000 habitants, and 8 deaths. Cabo Delgado reported 254 cumulative cholera cases with no cholera deaths. The number of cases declined dramatically due to prompt and appropriate control measures provided by the ministry of health (MoH), including adequate case management, implementation WASH interventions and a mass OCV vaccination campaign in both provinces [6].

**Current approach to cholera prevention and control in Mozambique:** responding to cholera represents an enormous burden for the national health service, education, housing and water resources, public service, among other sectors. The approach entails the creation of outbreak response coordination structures each time cholera is detected. The priority focus is the reduction of cholera mortality through the timely provision of emergency health care and WASH interventions. In general, these are a national multisectoral committee on health emergencies and a technical committee on cholera and other diarrheal diseases, with representation for all levels of the national health system, which are then mirrored at the provincial level, with corresponding terms of reference.

**Surveillance:** this is the first pillar of cholera prevention and control, since implementing any interventions requires detailed, accurate data based on epidemiologic and laboratory surveillance systems able to timely detect, confirm and monitor cholera occurrence. In Mozambique, cholera is one of the mandatory-reported diseases detected and reported through WHO Integrated Disease Surveillance and Response (IDSR). Unfortunately, gaps related to underreported data owing to lack of understanding of case definition, the absence of diagnosis capacity or culture of *Vibrio cholera* are frequent in the country [3]. Cholera can have a sudden and explosive onset. The substantial lull periods combined with the lack of sensitive surveillance systems (resulting from limited diagnosis capacity, poor data quality and lack of understanding the disease case definitions), have challenged the ability to detect and monitor cholera occurrence with high specificity and sensitivity. Symptoms can be indistinguishable from other causes of diarrhea. In addition, approximately 10-20% of the cholera cases are symptomatic present as mild acute watery diarrhea; and some patients may not seek for care when symptoms are mild [7]. As a result, cholera cases might not be suspected and reported. On the other hand, watery diarrhea cases caused by other enteric pathogens may falsely be reported as cholera cases when laboratory diagnosis by culture or polymerase chain reaction (PCR) are not available.

Cholera is prone to underreporting in Mozambique. Some decision makers may perceive that reporting cholera cases, or deaths could have a negative impact on their performance indicators. So sometimes cholera is put “hidden under the carpet”. Without effective and robust surveillance systems in place, governments and partners are forced to make critical decisions on public health interventions based on limited data. In Mozambique, surveillance data quality is usually a challenge, and data with the breakdown of cases by a municipality is not available [3]. The limited access to remote and rural communities present challenges to monitor diseases cases and the vital events occurring at the community level. In addition, in many rural settings a significant proportion of cholera deaths occur before patients reach the treatment facilities and are, therefore, not captured by the hospital-based surveillance systems. Cross-border transmission is also poorly addressed. Trained human resources to correctly use the case definition with high sensitivity and specificity and laboratory capacity availability is crucial for efficient cholera surveillance and disease burden estimation. After two massive cyclones, the experience demonstrates that high-quality epidemiological data plays an important role in prioritizing and identify the population at risk of epidemic cholera [8].

**Case management:** after cholera is detected, the immediate priority is to ensure that cholera cases are treated as soon as possible to minimize disability and death. Cholera is an easily treatable disease through prompt administration of oral rehydration solution (OSR). Effective case management also involve the treatment units (CTU)/centers (CTD) [2]. Those needs to be strategical established were population that can have easily access. NCP need to prioritize the mapping of hotspots areas to reinforce the training in clinical protocols and maintenance provide life-saving treatment to cholera patients. In addition, a focus on community health care workers could be critical strategy and since they can take treatment and prevention activities at community level.

**Water, sanitation, and hygiene (WASH):** despite progress in extending access to WASH through the millennium development goals, Mozambique has one of the lowest levels of clean water consumption globally. The country ranks 128^th^ in terms of access to improved water and sanitation sources out of 135 countries. In 2014, the percentage of the population that lived below the international standard of poverty (USD 1.9 per day) was 62.9%, higher than the sub-Saharan average of 41.1%. It is estimated that only 25% of the Mozambican population has access to adequate improved sanitation conditions [9]. According to the global climate index, Mozambique is ranking in the top ten countries most affected to weather events, with frequent massive floods, which cause destruction of sanitation infrastructure and water flows through soil contaminated by sewage that are heavily enriched with pathogens such as *Vibrio cholera*. The practice of open defecation is common in several parts of the country, being more evident in the “free” spaces of peri-urban areas.

**Oral cholera vaccine (OCV):** traditional interventions have focused on improving clean water supplies and sanitation, and these remain as long-term objectives. Pending the improvement of infrastructure, new cholera vaccines have been developed that offer the possibility of short to middle-term intervention to reduce the cholera burden. With the advent of safe and effective killed whole cell-based OCV, which replaced early injectable cholera vaccines abandoned by WHO in the late 1970s due to their poor performance, OCV has been reconsidered as a key component of the public health response to cholera. The WHO Strategic Advisory Group of Experts (SAGE) recommended the consideration of cholera vaccines in both endemic and epidemic regions as short-term intervention to reduce the rates of cholera. Currently, there are three WHO prequalified safe, inexpensive, easy to deliver, and effective OCVs, and available through a stockpile.

The global OCV stockpile was established in 2013 by WHO, with an initial stock of 2 million doses with funding support from multiple donors. OCVs have become a game-changer. They have been deployed for preemptive and reactive campaigns, and by taking two complete doses, the vaccine provides immunity for 2-3 years and increases the levels of herd protection among the population in the same catchment area. From 2013 to July 2017, 25 million OCV doses were requested by cholera affected countries (including Mozambique) and other agencies. Nearly 18 million doses (71%) were approved and around 13 million were shipped in 46 contexts, including humanitarian crises, outbreak response and endemic areas [10]. OCVs have been rapidly integrated into cholera prevention and control plans; however, the link with WASH investments remains limited. In Mozambique, previous experiences of conducting cholera mass vaccination campaigns during emergency response, have showed that OCV can be an effective and rapid tool to control cholera outbreaks [8]. However, vaccination can be an even more effective tool when it is used in endemic hotspots since it prevents the disease from transmitting further. Improving cholera surveillance to identify these cholera hotspots and guide planned (within the NCP) mass vaccination campaigns is key to achieve cholera elimination, especially when large scale vaccination is paired with longer term WASH measures.

**Community engagement:** community engagement is key to ensure long term changes in behaviour and to the control of cholera. In Mozambique, health promotion and community engagement are priority areas for the reduction of cholera morbidity and mortality. Mozambique has a history of changes in the political landscape, which have impacted the social determinants and acceptance for health interventions over the years. In the north of the country, episodes of violence have been associated with cholera outbreaks. Communities have attacked health workers accusing them to have poisoned the drinking water and spread the disease instead of preventing it. This unrest is often due to a simple misunderstanding, that the word for chlorine in Portuguese is “cloro”, which is very similar to “cólera” (cholera). These episodes of violence also result in the concealment of cholera cases and deaths from the health authorities, and in unsafe burial practices. The NCP needs to address the specific local context of cholera transmission in hotspots, and focus on strengthening health education for appropriate interpersonal, social and behavioral change to support long-term prevention and control at the community level. Health education campaigns, adapted to local culture and beliefs, should promote the adoption of appropriate hygiene practices such as handwashing with soap, safe preparation and storage of food and safe disposal of the feces of children. Funeral practices for individuals who die from cholera must also be adapted to prevent infection among attendees.

**Leadership and coordination:** as a response strategy to curb recurrent cholera outbreaks, MoH established action plans against cholera and organized the multi-sectoral cholera elimination plan group for the development of the NCP, ensuring strong commitment, an effective interinstitutional coordination process and the mutual involvement of all sectors and partners. The MoH and its partners have been triggering outbreak mitigation measures annually, before, and during the critical period that usually coincides with the rainy season or flood periods, from December to June. The MoH is leading the development of the cholera control plan with the involvement of other sectors, including water, education, and sanitation, through multi-sectoral coordination platforms and a cholera task force. However, determining the effectiveness of these coordination structures and the engagement of other sectors as water is likely to be a challenge due to competing priorities, lack of coordination mechanisms, resources, and time. Understanding the roles, priorities, and measures of success of different sectors is vital in driving more joined-up approaches to a successful implementation plan.

**The need of holistic vision:** most, if not all, the tools to eliminate cholera are available in Mozambique. However, developing a sustainable NCP does not only require technical solutions, but a holistic vision that can effectively foster a synergy between all actors involved according to a multisectoral approach that goes beyond health. First and foremost, the approach should be founded in an effective cholera surveillance system across the country. Cholera burden data is critical to decision-making in public health interventions based on concrete evidence. Unfortunately, in Mozambique, the cholera disease burden is poorly understood since a substantial number of cases are not reported due to the limited capacity of the surveillance system and obstacles in reporting.

Cholera can be eliminated with sustainable long-term comprehensive and sustainable solutions such as access to water and sanitation facilities, and good hygiene practices (WASH interventions). However, despite the large evidence on the impact of WASH interventions, these are costly and require long-term investment and political commitment [5] and in Mozambique, this could be unrealistic. On the other hand, some strategies can be easily implemented in the cholera hotpots areas, such as community action to end open defecation, construction of latrines, ensure the provision of point-of-use of safe water, enhanced water-quality testing, and monitoring, encouraging household water treatment with sodium hypochlorite, manage fecal waste, increase the access, and use of handwashing facilities. The Mozambique plans have focused so far more on short term solutions and control measures during emergency situations such as outbreaks and neglect longer-term approaches and critical infrastructures for water supply and sanitation in the plan. WASH is considered the most effective solution to eliminate at least 50% of the disease in the sub-Saharan African region [1]. Similarly, the use of OCV is still too skewed on emergency response, rather than on conducting mass vaccination campaigns to reduce the burden in cholera hotspots. Those two strategies need to be integrated in a multi-sectoral comprehensive plan for effective cholera control and prevention. Comprehensive cholera control and prevention strategies are cost-effective in long term. However, without adequate political and financial commitment to the NCP, progress is impossible. As it is without putting in place mechanisms that ensure governance, coordination, planning, monitoring, financing, and accountability.

**Conclusion:** cholera is an old disease that could be solved with relatively simple interventions, integrating health measures, with ensuring access to water, sanitation, and hygiene. The country must have a clear vision and strategy of challenges and opportunities for the development of NCP with a longer-term vision toward cholera control or elimination. The plan needs to address a comprehensive approach, including strengthen disease surveillance system with laboratory support, clinical care, the use of OCV both in emergency and non-emergency situations, provision of safe water and sanitation, social and behavioral change and identify the populations most at risk for cholera to better target hotspots for improvements in long-term sustainable intervention as WASH and OCV. Investment in public WASH infrastructure should be a priority in areas of high risk of infection to prevent cholera and other diarrheal disease outbreaks. As a high-burden cholera endemic, Mozambique government and needs to elevate cholera as a public health priority and invest in a long-term preventative approach. To achieve this, there adequate involvement and political commitment at the highest levels of the government will be paramount. Last but not least mechanisms for funding and accountability, across all relevant sectors should be established.
